# Thermoplastic Composite Hot-Melt Adhesives with Metallic Nano-Particles for Reversible Bonding Techniques Utilizing Microwave Energy

**DOI:** 10.3390/polym16243496

**Published:** 2024-12-15

**Authors:** Romeo Cristian Ciobanu, Mihaela Aradoaei, George Andrei Ursan

**Affiliations:** Department of Electrical Measurements and Materials, Gheorghe Asachi Technical University, Boulevard D. Mangeron 71, 700050 Iasi, Romania; mosneagum@yahoo.com (M.A.); andrei_urs@yahoo.com (G.A.U.)

**Keywords:** nano-composites, hot-melt adhesives, reversible bonding, microwave technology

## Abstract

This study investigated the creation of nano-composites using recycled LDPE and added 7.5 wt% nanofillers of Al and Fe in two varying particle sizes to be used as hot-melt adhesives for reversible bonding processes with the use of microwave technology. Reversible bonding relates to circular economy enhancement practices, like repair, refurbishment, replacement, or renovation. The physical–chemical, mechanical, and dielectric characteristics were considered to determine the impact of particle size and metal type. Through the investigation of electromagnetic radiation absorption in the composites, it was discovered that the optimal bonding technique could potentially involve a frequency of 915 MHz and a power level of 850 × 10^3^ W/kg, resulting in an efficient process lasting 0.5 min. It was ultimately proven that the newly created hot-melt adhesive formulas can be entirely recycled and repurposed for similar bonding needs.

## 1. Introduction

Bonding technologies are commonly used in various industries, particularly in sectors like automotive, naval, and aerospace, where composite materials, metals, and plastics are employed. Such materials are commonly joined during the manufacturing processes with mechanical fasteners, welding, or adhesive bonding techniques. The first process is slow, costly, labor-consuming, and sometimes non-esthetic. The latter two processes have many benefits over mechanical fastening, such as a faster process speed, weight-saving properties, less material demand, and cost effectiveness. These techniques are currently increasingly used for assembling processes; however, they have the most important drawback of being irreversible. Disbonding or, more advantageous, reversible bonding are new ideas connected to circular economy, primarily driven by the European Community’s recycling plan in different industries, such as the automotive sector, which follows the End-of-Life Vehicles Directive [1]. Reversible bonding may be necessary to separate a temporary structure or a previously bonded assembly for activities such as repair, refurbishment, replacement, or renovation. Streamlined disbanding processes also make it easier to recycle materials and components from items and structures that are bonded with adhesive. Salvageable components must be recovered without any harm in bonded composite structures for successful debonding. Frequently, saving medium-cost components can be profitable in order to decrease assembly or lead time expenses. After removing the components, one can proceed with reattaching or reassembling them so that the bonded product can be put back into use.

Nowadays, hot-melt bonding techniques play a vital role with a relevant market impact (the hot-melt adhesives industry is projected to grow from USD 10.07 billion in 2024 to USD 13.42 billion by 2032 at a CAGR of 4.33% during the forecast period) [1]. Hot-melt adhesives, which are polymer-based glues that are applied in a molten state, change from a liquid state at high temperatures to a solid state at low temperatures over a small temperature range. Once the hot melt cools down, it becomes solid and creates a sticky connection between two selected surfaces [2]. Hot-melt adhesives are very beneficial for mass production, with low expenses due to ease of application, quick bonding, and the use of a single component without needing additional materials or catalysts for bonding. Hot melts have the ability to stick to a range of materials, such as plastics, paper, wood, metal, and fabrics, allowing for diverse application options. They are considered environmentally friendly since they do not include solvents or volatile organic compounds (VOCs), decreasing their impact on the environment. Unlike solvent-based adhesives, hot melts present lower health hazards, too [3]. The reversible bonding techniques involving hot melts need to employ approaches that can be categorized based on the energy types involved and how the bonding is disrupted. In order to break the connection created by a regular hot-melt adhesive, the bond must be heated to a temperature higher than the adhesive’s melting point. In reality, it is frequently not feasible to administer enough heat to a sizable bonded unit or to bonded components that are sensitive to temperature. Determining how easy the hot melt is to remove depends on the method of removal chosen, the time taken for removal, and the equipment used. It is important to choose a method that requires minimal disassembly time in order to prevent extended downtime.

Traditional methods of bonding with hot melt can involve thermal, electrical, or laser processes used alone or together. Thermal processes consist of exposing materials to heat from sources like pressurized steam or hot air, either directly or by using hot plates that come into contact with the surfaces being bonded [4,5]. Certain drawbacks are clear and restrict the application of these thermal techniques: ununiform heating of surfaces leading to ununiform bonding, inducing internal mechanical residual stress or deformation, collateral effects related to the difference of dilatation—with limitations in performance, insufficient heat resistance, limited bonding strength, high energy consumption, and a longer bonding process time. The hot melt may not be adequately heated, which affects not only the bonding strength but also the efficiency and quality of the debonding process. Laser bonding involves laser treatment with UV and IR radiation of the surfaces to be bonded, but it has low efficiency and no relevant industrial applications because it is normally associated with thermal bonding [6]. Finally, adhesive bonding technology activated by electromagnetic fields is innovative and an alternative to the thermal bonding technologies used today in manufacturing sectors. The main advantage lies in the heating of the hot melt before heating the bonding surfaces, resulting in the hot melt being sufficiently heated.

**Figure 1:**
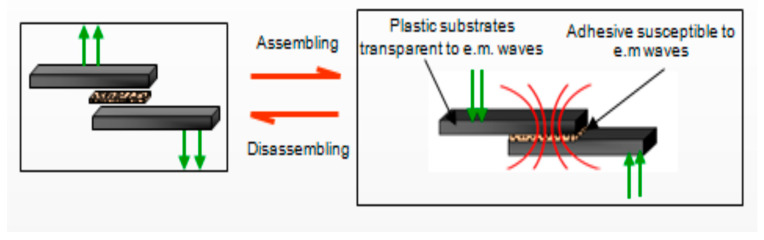
Assembling/disassembling process under electromagnetic field (marked in red).

There are three main directions of electrical bonding: by thermal effects of conduction in direct current (direct resistance heating and/or partial discharging), as described in [7,8,9,10,11], which is associated with electrochemical effects at the interfaces [12]; induction bonding under alternating field, as described in [11,13,14,15,16,17,18,19], which exploits either the dielectric or hysteresis properties of adhesives mainly under 400 kHz frequency; and, finally, a newer approach of using microwave exposure at frequencies exceeding 1 GHz [20,21,22]. The challenge of these technologies is to develop new multifunctional and highly reliable hot-melt materials and related processes capable of providing a strong and energy-efficient joint, which can be reversed to disassemble the components by a specific trigger of an electromagnetic field. The formulation of innovative hot-melt adhesives manufactured with functional micro/nanosized fillers, opportunely functionalized depending on the adhesive matrix used, may lead to an increase in the mechanical performance in terms of the intrinsic mechanical resistance of the adhesive, improvement of the mechanical performance of the joints (shear stress, flexural stress, and compression stress of joints), and improvement of adhesion to different typologies of substrates, but it also assures an increased heating rate by applying electromagnetic fields. Depending on the heating principle to be used (dielectric or hysteresis) and to the frequency to be applied, different functional particles can be employed, e.g., conducting particles (graphite or metallic powders, carbon nano-tubes, etc.), or particles with ferritic properties (iron oxides, nickel oxides, etc.); in rare cases, conducting polymers may be additionally introduced [15,18,19,21,22]. The amount of the heat generated depends on the nature, percentage and morphology of the particles, and the dissipated heat is able to quickly reach the melting temperature of the thermoplastic polymeric matrix in order to activate the adhesive for a rapid assembling or disassembling process, as presented in Figure 1.

The hot-melt adhesives formulation can also include oligomers, plasticizers, etc., which exhibit polar properties, adding new advantages to the bonding process under an electromagnetic field. It is beneficial for the hot-melt adhesive if the oligomers that coordinate ionic species are able to mix well in their molten state with functional particles, acting as a supplementary tackifier for the adhesive composition [23,24,25,26,27,28,29,30,31].

Recent research has focused on evaluating the environmental impact of electrically activated hot-melt adhesives, highlighting their lower impact during manufacturing and reduced energy requirements compared to thermal processes [32,33]. There is potential for further improvement by implementing innovative materials and electromagnetic equipment to increase efficiency and productivity and to decrease electricity consumption during application.

Currently, there is no research on utilizing MW-activatable adhesives with functional particles, and the study presented shows that incorporating metal particles into thermoplastic formulations to make MW-activatable adhesives reduces energy consumption by allowing for a quick activation time of only a few seconds. This paper addresses, with priority, the development of polyethylene-based hot-melt adhesives due to their various benefits, such as excellent life stability, better thermostability, a wider bonding range, and lack of tendency to char or to create unpleasant smells. In 2018, the European Commission approved a European plastics strategy as a component of the EU’s circular economy action plan [34]. The objective was to aid, enhance, and speed up the execution of actions to decrease plastic waste. Another important goal is the mandate that by 2030, all plastic packaging sold in the EU must either be reusable or economically recyclable [35]. For such reasons, the MW-activatable adhesives manufacturing used a polyethylene matrix from recycled sources. In this way, the innovation involves creating new adhesive formulations that are eco-friendly and can be fully recycled and reused for similar bonding needs.

## 2. Materials and Methods

### 2.1. Materials

Composites containing recycled LDPE powder as the polymer matrix incorporated nanofillers of Al and Fe in two different particle sizes at the nanoscale: 50 nm and 800 nm. The main characteristics of the spherical-shaped metallic powders of Al and Fe and their dimensional distribution are presented elsewhere [36]. It was shown that the variance in the particle dimensions (larger for Fe particles) benefits adhesive manufacturing because the larger distribution lowers the particle costs by approximately tenfold compared to narrower distributions and offers increased conductivity and superior dielectric loss—dielectric characteristics expected with higher values for MW-activatable adhesives.

**Figure 2:**
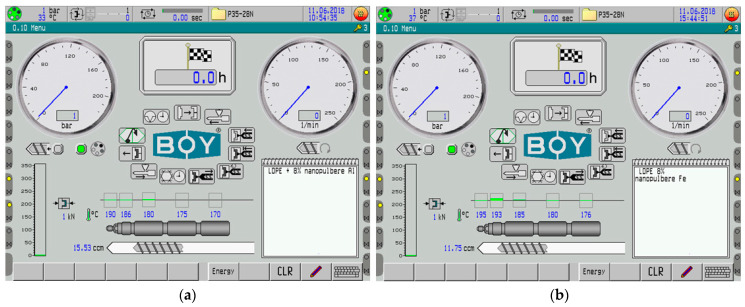
The control monitor of the injection machine for composites containing (**a**) Al and (**b**) Fe.

To produce the samples for the experiments, the polymer and the nano-conductive powder were mixed together for 15 min in a TURBULA T2F cylindrical mixer from Artisan Technology Group (Champaign, IL, USA). The mixer has a 1.3 L capacity mixing basket and a rubber-ring-based clamping device, with a rotation speed of 40 rpm. Finally, the composite materials were produced using the Dr. Boy 35A injection machine (Dr. Boy GmbH & Co. K, Neustadt-Fernthal, Germany) with a screw diameter of 28 mm, L/D ratio of 18.6 mm, calculated injection capacity of 58.5 cm^3^, maximum material pressure of 2200 bar, and minimum real injection capacity of 500 mm. In all the experimental models, a 3% ratio of compatibilizing agents was employed, including Poly(ethylene glycol) methacrylate at 1%, an Ethylene Acrylic Acid Copolymer at 1%, and Tegomer^®^ E 525 (Evonik Operations GmbH, Essen, Germany) at 1% (wt%). Table 1 shows the optimal temperature range for the injection cylinder’s five heating zones during the injection process for LDPE composites containing Al and Fe. Figure 2 shows the control monitor with the temperatures set for the five heating zones of the injection machine for the Al- and Fe-containing composites. Slightly higher temperatures for the Fe-containing composites were needed. The recipes’ descriptions are given in Table 2. The optimal value of 7.5 wt% metallic powder was determined after an extended simulation using specialized software for S parameters and absorbed microwave energy in polymeric nano-composites containing 5–10 wt% iron and aluminum powder of two particle sizes within the frequency range of 0.1–3 GHz.

### 2.2. Characterization Methods and Related Equipment

(i) Electron scanning microscopy SEM was performed with a field emission and focused ion beam scanning electron microscope (SEM), model Quanta FEG 250, with STEM and EDX detectors (Thermo Fisher Scientific Inc., Waltham, MA, USA). The analysis method was LowVac, with water vapor, which does not allow the samples to be damaged. The SEM charging occurrence was significantly reduced due to the low vacuum in the specimen chamber of the SEM.

(ii) A Netzsch STA PC 409 thermal analyzer (Erich NETZSCH B.V. & Co. Holding KG, Selbwas, Germany) was used for thermogravimetric analysis. The working atmosphere was synthetic air, 100 mL/min in alumina crucibles. The heating program was 35–1200 °C, with a heating speed of 10 °C/min.

(iii) The hydrostatic density was determined utilizing an XS204 Analytical Balance (Mettler-Toledo, Columbus, OH, USA), characterized by the following specifications: maximum capacity of 220 g, precision of 0.1 mg, linearity of 0.2 mg, internal calibration, equipped with a density kit for solids and liquids and an RS 232 interface (Mettler-Toledo, Columbus, OH, USA). The measurements were conducted at a temperature of 21 °C, with three consecutive repetitions, and the error was calculated. The density was determined as the mean value between the three consecutive repeated measurements.

(iv) Shore hardness measurements were taken with a common Shore “D” digital durometer, as the mean of 5 measurements.

(v) The equipment for determining the mechanical features was a specialized PC-controlled universal tensile testing machine (Qiantong, China), with nominal force: min 20 kN, allowing measurement of tensile strength and elongation.

(vi) Microindentation tests were performed with the use of a compact open platform equipped with a Nano/Micro Indentation Tester and a Micro Scratch Tester, from CSM Instruments SA, Peseux, Switzerland. The mechanical tests were performed at ambient temperature, as 5 measurements on each sample, with the average of the obtained values and their standard deviation being reported.

(vii) The degree of swelling was determined by measuring the variation in the mass of the samples at predefined immersion intervals, utilizing the XS204 Analytical Balance.

(viii) The dielectric properties were carried out using a Broadband Dielectric Spectrometer (Novocontrol GMBH, Montabaur, Germany) encompassing an Alpha frequency response analyzer and a Quattro temperature controller, with tailored measurement cells up to 40 GHz.

## 3. Results and Discussion

### 3.1. SEM and X-Ray Fluorescence (XRF)

**Figure 3:**
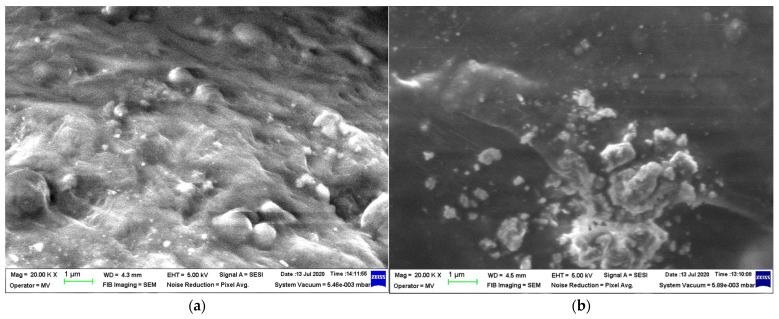
SEM images for Al-containing composites: (**a**) M1 and (**b**) M2.

**Figure 4:**
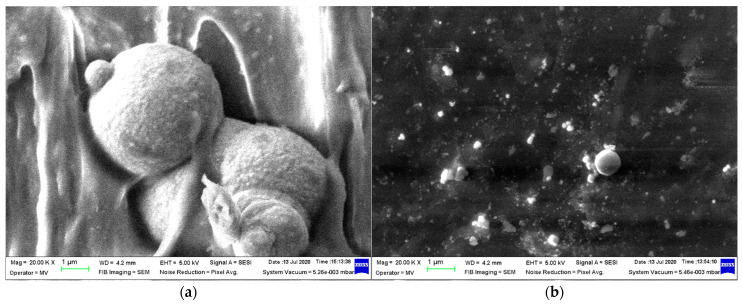
SEM images for Fe-containing composites: (**a**) M3 and (**b**) M4.

Micrographs were taken at 20,000 magnification to assess how the metallic particles were incorporated into the LDPE matrix in the produced composite materials. The examination of the compositions shows that Al, depicted in Figure 3, is associated with smooth powder particles, while Fe, shown in Figure 4, is linked to rough spherical particles in both size dimensions. However, it was observed that the Al particles behaved differently compared to the Fe particles, as they are mostly exposed rather than being covered by the polymer matrix, showing a stronger connection. Choosing different additives for the compatibility of composite components can enhance the compounding process of aluminum. Furthermore, it has been observed that the most uniform samples are produced with 50 nm particles, regardless of their metal type, especially for M4, where the dispersion of Fe particles is technologically optimized.

**Figure 5:**
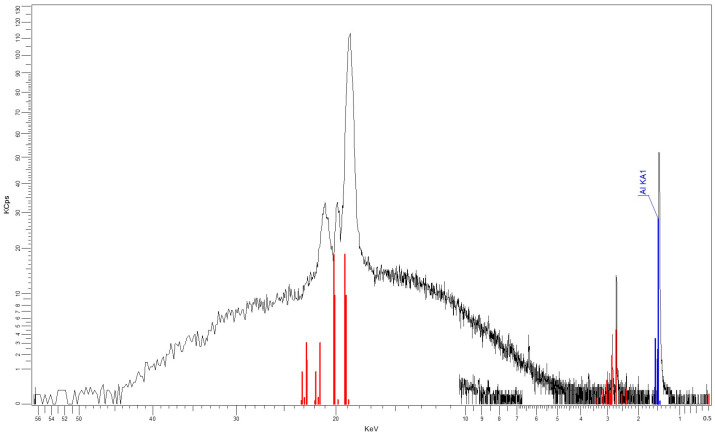
XRF spectrum for M1.

**Figure 6:**
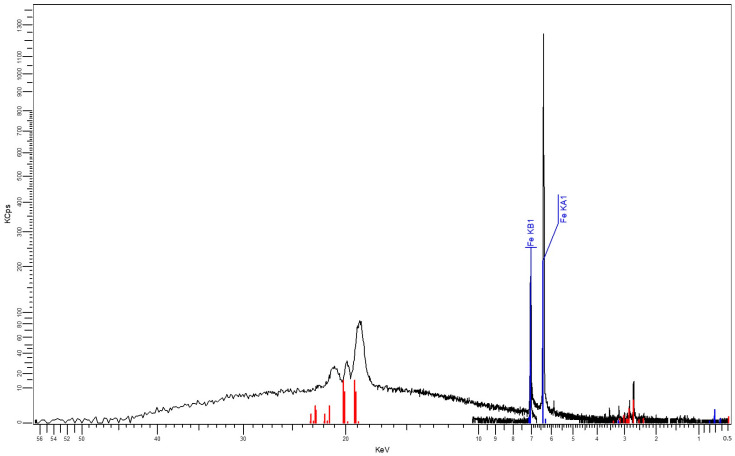
XRF spectrum for M3.

The results obtained as XRF characteristics, shown in Figure 5 and Figure 6, confirm the nature and percentages of the nano-powder in the composite materials, as described in Table 3 and Table 4.

### 3.2. Results Obtained from Micro-Indentation Tests

Table 5 presents the results of H_IT_, HV, E_IT_, S, W_elastic_, W_plastic_. W_total_ and η_IT_ obtained from the microindentation tests for the composite samples tested, as presented in ASTM E2546-15 with the Oliver–Pharr calculus method [37]. The mechanical characteristics, mainly, indented hardness H_IT_ and Vickers hardness HV, are slightly higher for the composites containing smaller metallic particles, regardless of the metal type. The highest values were reached by M4. A reduced modulus of elasticity E_IT_ was found to be identical for all the samples. The elastic indentation work W_elastic_ and reversible elastic deformation work show higher values for samples with larger-sized powders, with slightly increased values for composites containing Al, e.g., M1. As regards the mechanical plastic deformation of indentation W_plastic_, slightly increased values were found for composites containing larger-sized Fe powder, e.g., M3. The examination showed distinct variations in the sample structure on the surface, linked to the type and size of metallic additives and their impact on the interaction with the matrix during compounding. These results will be connected to other characteristics for further discussion, but initially, the composites containing aluminum appeared to have a more flexible surface compared to those containing iron.

### 3.3. Results Obtained from Density Analysis

The hydrostatic density results from Table 6 show close values, which can be attributed to the high polymer content. In theory, if the metal powder content is consistent in all the samples, the discrepancy may be attributed to the variance in atomic mass between aluminum and iron, resulting in a clear difference in density between M1 and M3, or M2 and M4. However, we also observed variances between M1 and M2, as well as between M3 and M4, despite containing identical metallic particles, possibly due to the way the particle size influences the composite structure. The composites containing 50 nm particles may have a lower density because of the looser connection between LDPE matrix elements, which is a result of the more evenly spread particles. This supports the findings of the SEM analysis.

The composite material M3 had the greatest density, while M2 had the smallest when compared to the other composite materials acquired.

### 3.4. Results Obtained from Shore Hardness Tests

The shore hardness was determined in accordance with ASTM D2240-00 [38], and the results are displayed in Table 7.

The hardness of samples M2 and M4 was higher than that of M1 and M3, respectively, because the metallic particles with a 50 nm dimension are better dispersed, resulting in lower surface roughness. The slightly elevated values of the Fe-containing composites in comparison to the Al-containing composites may be attributed to the increased attraction of Fe particles to the polymer matrix, leading to a more robust bond between the polymer and particles, as supported by the SEM analysis results.

### 3.5. Results Obtained from the Mechanical Tests

The mechanical tests were carried out according to the SR EN ISO 527-2:2000 standard [39], on five samples each. The statistical interpretation of the results consisted in determining the average value of five measurements, excluding values outside the range, with a confidence level of 95%, Table 8. Once the metallic powder was added to the polymer matrix, the composite material became more rigid, showing a rise in mechanical strength but a decrease in flexibility and elasticity. The resistance increase was greater, while the elongation was lower in the Fe-containing composites and overall, in the composites with smaller particle dimensions that are more evenly distributed. Therefore, M4 had the highest mechanical resistance value, while M1 had the lowest. In terms of flow resistance, materials with larger particle dimensions were favored, such as M1, and the type of metal had little impact upon flow resistance.

### 3.6. Results Obtained for the Degree of Swelling

**Figure 7:**
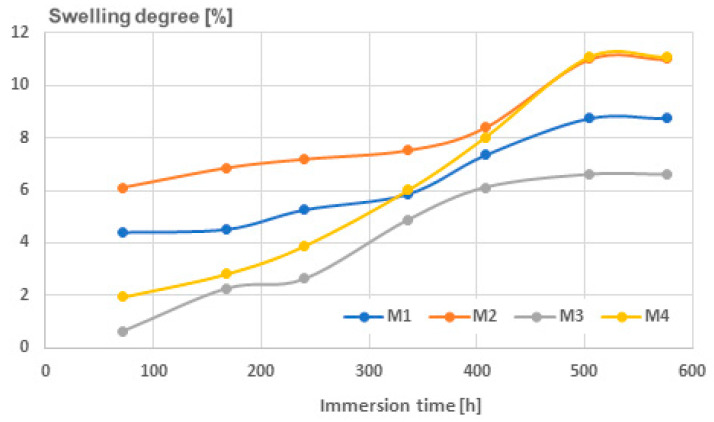
Swelling degree for water.

The procedure was carried out according to SR EN ISO 175/2011 [40], and the results are presented in Table 9, for immersion in water, and in Table 10, for immersion in solvent, here, toluene. Essentially, mixing metallic nano-particles with polymer matrices can create micro-voids during the melt process, affecting the surface roughness of the samples and determining liquid attraction and insertion potential. With regards to water absorption, as shown in Figure 7, it was observed that the swelling degree remained low until 400 h of immersion, after which it rapidly increased until reaching a saturation point at around 600 h. The level of swelling was greater in the composites with Al, as well as in the composites with smaller particles because they have more micro-voids in the materials. After 400 h of immersion time, the composites with smaller particle dimensions exhibited an intriguing phenomenon where the swelling degree appeared to be nearly identical, regardless of the type of metallic particle present.

**Figure 8:**
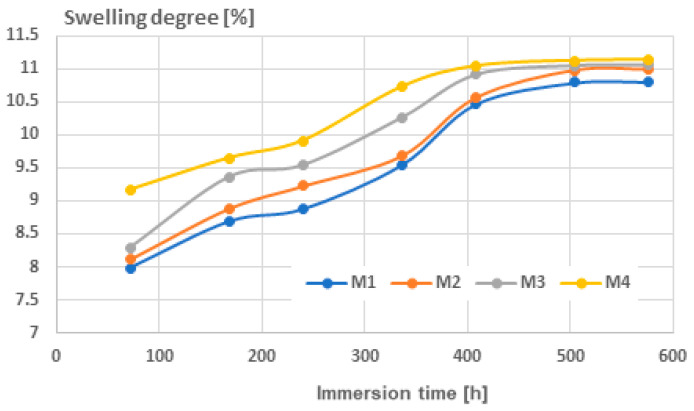
Swelling degree for toluene.

In relation to the level of swelling with toluene, shown in Figure 8, it was observed that because of toluene’s attraction to the matrix, the values were elevated even with shorter immersion times. The overall trend holds true: the composites with aluminum had a greater degree of swelling, as did the composites with smaller particles. For the solvent, saturation happened sooner, around 450 h, and the degree of swelling was similar for all the composites, showing that toluene absorption is not greatly affected by the number or size of micro-voids. A noteworthy observation was made about the ultimate swelling saturation levels in water and toluene, which were nearly identical at around 11%, specifically for samples M2 and M4. This indicates a total filling of the micro-voids, which are evidently more abundant and smaller in size compared to M1 and M3.

### 3.7. Results Obtained from Thermal Analysis

**Figure 9:**
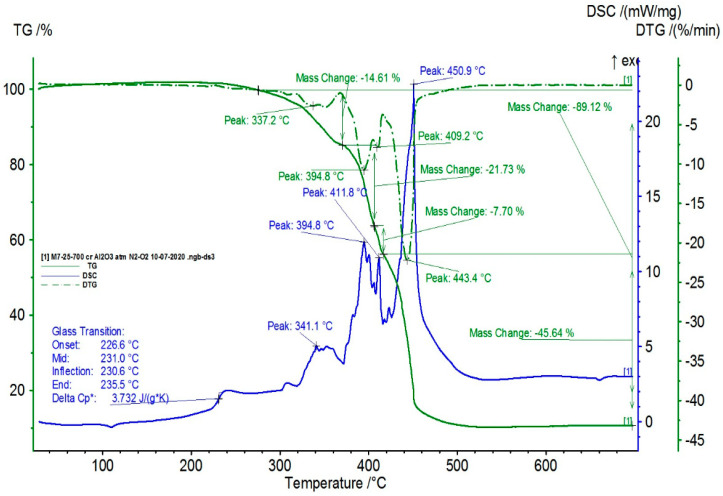
Thermal characteristics obtained for M1.

**Figure 10:**
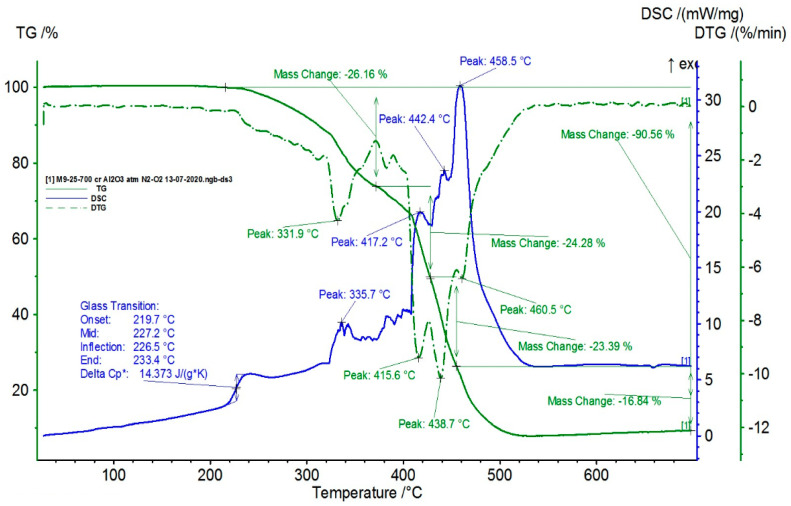
Thermal characteristics obtained for M2.

**Figure 11:**
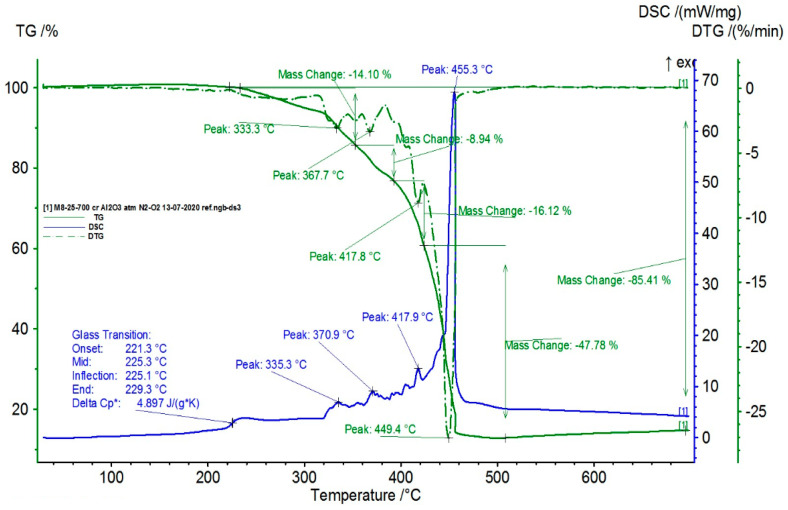
Thermal characteristics obtained for M3.

**Figure 12:**
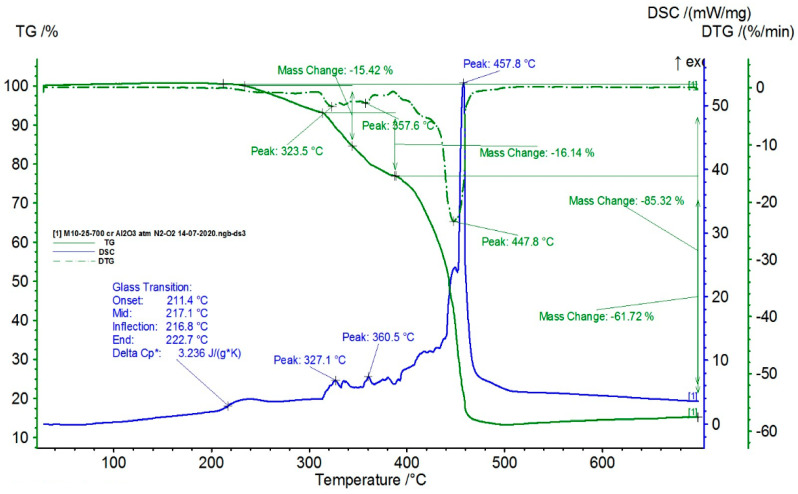
Thermal characteristics obtained for M4.

The TG/DSC characteristics of the composite samples are presented in Figure 9, Figure 10, Figure 11 and Figure 12.

From the statistical interpretation of the obtained results, it was found that for the composite materials with the LDPE polymer matrix, the melting temperatures were very close due to the majority concentration of the base polymer, varying in the range of 103–106 °C—the specific melting temperature of LDPE. It was noticed that the composites with lower-dimension metallic particles presented slightly higher melting temperature values. The respective results are in line with similar findings in [41,42,43,44]. The temperatures of the start of the first oxidation process (OOT_1_) varied in the range of 190–215 °C, and the temperatures of the start of the second oxidation process (OOT_2_) varied in the range of 250–315 °C, Table 11. Heating further led to thermo-oxidative processes until complete destruction of the polymer matrix. Other specific values of the DSC parameters are presented in Table 11. It was noticed that the composites with lower-dimension metallic particles presented slightly lower values of oxidation processes.

### 3.8. Results Obtained for Dielectric Properties

**Figure 13:**
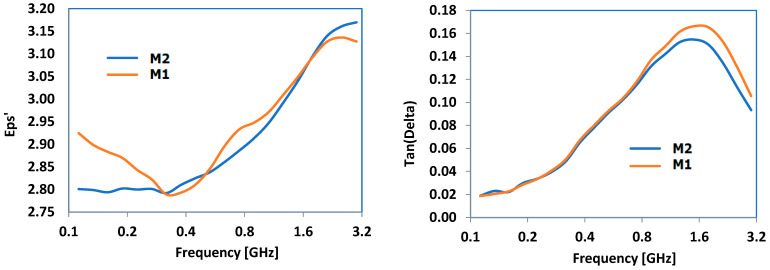
Dielectric properties of Al-containing composites.

**Figure 14:**
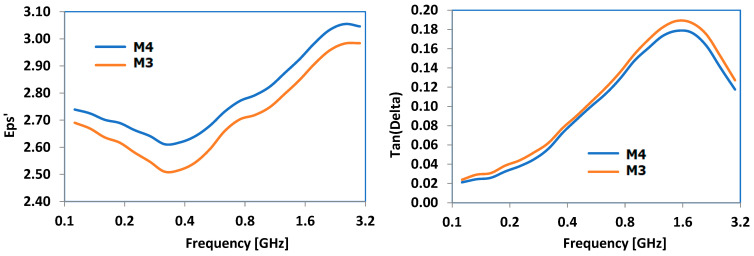
Dielectric properties of Fe-containing composites.

The primary dielectric properties that were examined included dielectric permittivity (eps’) and the dielectric loss factor (Tan(Delta)). The frequency domain was selected based on the main frequencies used in industrial microwave technologies. Figure 13 illustrates the findings for the composites containing Al, while Figure 14 demonstrates the results for the composites containing Fe. Regardless of the type of metal, there was a consistent increase in both characteristics versus frequency, beginning at 0.8 GHz and peaking at around 1.4 GHz for Tan(Delta) in every scenario. Particle size had a minimal effect, even though it appears that compounds with larger particles may have shown slightly increased parameters values across all the frequency ranges, regardless of the metal type. Considering that Tan(Delta) is a critical factor in converting microwave energy into heat through dielectric loss, the frequency range of 0.9–1.7 GHz is the most effective for such materials.

### 3.9. Results Obtained for Electromagnetic Radiation Attenuation

**Figure 15:**
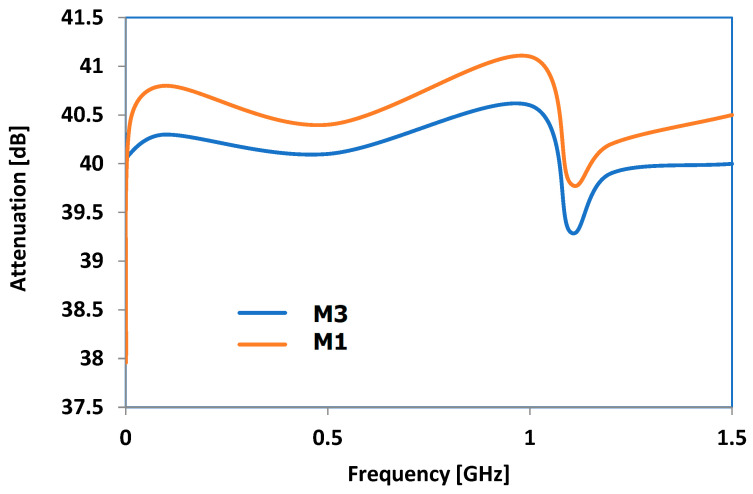
Comparative electromagnetic radiation attenuation of M1 and M3 composites.

The dielectric properties obtained need to be verified by examining how electromagnetic radiation is absorbed in materials, which is a crucial factor for how well they function as hot melts, as they should retain as much microwave energy as they can. Figure 15 shows the features of M1 and M3, which are considered the most suitable as a recipe according to the previous observations. The attenuation reached a peak at approximately 0.9 GHz, while showing a unique shape at frequencies above 1 GHz, resulting in reduced attenuation levels. The sharp decrease found around 1.15 GHz is specific to microwave waveguides, where the peculiar spacing of particles favors the transmission of microwaves through the material within a narrow frequency range. Considering all the findings from studying microwave effects, it can be determined that the optimal frequency range for maximizing hot melt usage is 0.9–1 GHz. Thankfully, there are currently large industrial/commercial microwave ovens utilizing 915 MHz, making it possible to develop bonding technologies using the achieved composites as hot melts at the same frequency.

### 3.10. Tests upon the Feasibility of Reversible Bonding Techniques Utilizing Microwave Energy

**Figure 16:**
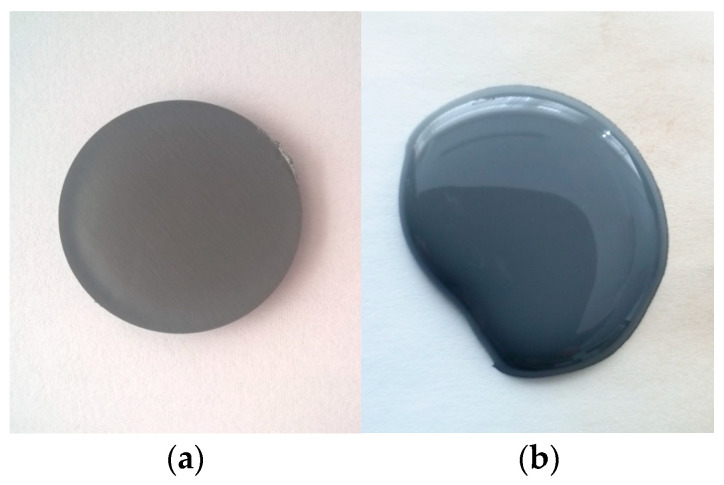
Images of hot-melt composite M1: (**a**) before exposure and (**b**) after exposure to microwaves.

Experiments were conducted to test the feasibility of bonding methods using microwave energy at 915 MHz, with varying emission power levels ranging from 100 to 1000 × 10^3^ W/kg. Figure 16 displays a sample subjected to microwave heating, exhibiting a slight change in color at elevated temperatures, attributed to the aluminum’s coloring effect.

**Figure 17:**
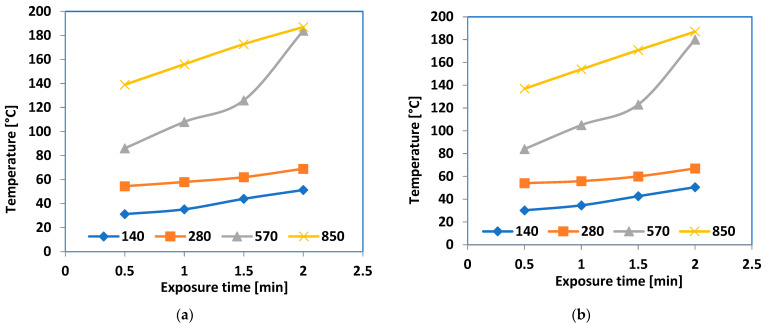
Heating characteristics at different power values (10^3^ W/kg) of microwave exposure for hot-melt composites (**a**) M1 and (**b**) M2.

**Figure 18:**
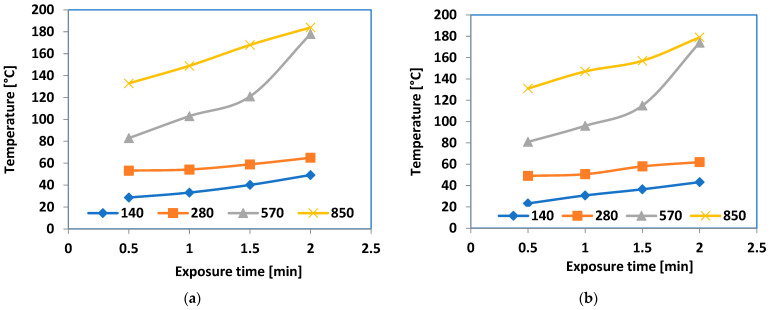
Heating characteristics at different power values (10^3^ W/kg) of microwave exposure for hot-melt composites (**a**) M3 and (**b**) M4.

Figure 17 shows the heating properties of microwave exposure on the Al-containing composites at different power levels (140, 280, 570, and 850 × 10^3^ W/kg), while Figure 18 represents the same for the Fe-containing composites.

Upon initial observation, it became clear that energy levels below 400 × 10^3^ W/kg could not adequately raise the temperature of the composites to a significant degree within a reasonable timeframe, with the temperature not exceeding 60 °C. The effectiveness of 570 × 10^3^ W/kg power utilization was achieved after the samples had been exposed for over 1.5 min, regardless of the type of sample. When a power of 850 × 10^3^ W/kg was used, the efficiency of the process increased, even with lower exposure times, starting from 0.5 min. A notable phenomenon was noticed, i.e., the temperature reached after 2 min of exposure was quite similar at both 570 and 850 × 10^3^ W/kg powers for all the samples. This occurrence is not caused by an electromagnetic impact but by a distinct effect in the melted composites, as 180 °C represents a high fluid state of the composites, regardless of their receipt, a temperature that should not be reached in bonding technologies utilizing this type of hot melt.

**Figure 19:**
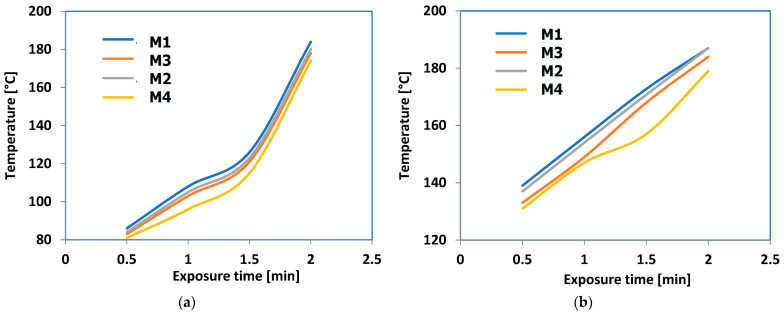
Heating efficiency of microwave exposure for hot-melt composites (**a**) at 570 × 10^3^ W/kg power and (**b**) at 850 × 10^3^ W/kg power.

A more in-depth evaluation was conducted on the actual heating efficiency of microwave exposure for the hot-melt composites, at 570 × 10^3^ W/kg power compared to 850 × 10^3^ W/kg power, as shown in Figure 19.

The optimal temperature for the hot-melt composites to become functional for bonding processes was considered approximately 5 °C higher than the melting point of LDPE composites, which is about 135 °C. This reduced application temperature represented a significant benefit of the hot melts examined compared to traditional hot melts used in thermal processes, such as thermoplastic rubber around 200 °C and polyurethane or polyamide exceeding 250 °C.

Based on Figure 19, regarding the exposure time when the temperature level of 140 °C was reached, it took over 1.5 min at 570 × 10^3^ W/kg, and only 0.5 min at 850 × 10^3^ W/kg. Accordingly, the energy needed for bonding activities was about double when operating at 570 × 10^3^ W/kg, with the efficiency being roughly three times lower when compared with operating at 850 × 10^3^ W/kg. In present hot melt uses, a 0.5 min exposure is appropriate and may be equivalent to the typical thermal method with a similar duration. The M1 formula is suggested as the most efficient hot-melt option available. In this situation, it is not necessary to use metallic powders smaller than 800 nm or with limited dimensional range, or in higher amounts within composites. This provides a significant cost advantage, as powders sized 50 nm are at least five times more costly than those sized 800 nm.

**Figure 20:**
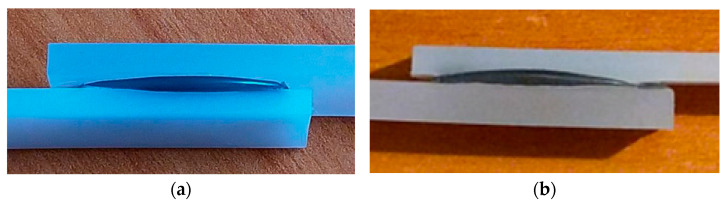
Examples of bonding configuration: (**a**) LDPE + LDPE/M1; (**b**) PP + PP/M3.

An illustration of the bonding testing is shown below, where Figure 20 depicts the bonding arrangement as a longitudinal joint of the samples by overlapping them at the ends. The mechanical tests were carried out according to the SR EN ISO 527-2:2000 standard [39], on five samples each. The overlap was of a length of three times the width of the items to be joined. The statistical interpretation of the results consists in determining the average value of five measurements, excluding values outside the range, with a confidence level of 95%. For the preliminary study, based on the materials typically used for bonding in the automotive industry, the main focus was on testing the bonding of low- and high-density polyethylene (LDPE and HDPE) and polypropylene (PP), with the results shown in Table 12. The test was conducted using the M1 and M3 hot melt samples, with the thickness being roughly 20% of the bonded item’s thickness, applying a pressure of 200 kPa, a minimal value needed to counteract the separation for the plastic items. The exposure was 0.5 min at 850 × 10^3^ W/kg. In every instance, a curing duration of approximately 1 min was noticed when the hot melt temperature dropped below 70 °C. A high cohesion of materials was observed in all the cases, but the bonding strength relies on the type of material and the hot melt composition. The peak value was reached with the LDPE + LDPE/M1 setup, while the lowest value occurred with the PP + PP/M1 configuration. The minimum elongation occurred with the PP + PP setup for both types of adhesives. The efficiency of the M1 formula was once again demonstrated.

Overall, microwave bonding offers considerable benefits compared to traditional thermal polymer-to-polymer bonding methods. The heating procedure initiates from the inside to the outside of the hot melt and is consistent, not impacting the materials to be joined, even regarding expansion or internal mechanical stresses. The energy consumption is greatly reduced, and the microwave bonding process efficiency may be finally improved by using a green electric supply, hence reducing CO_2_ emissions [45,46]. Conversely, the equipment size and complexity are minimized with microwave technology, making it readily suitable for robotic and remote operation. Furthermore, reheating and subsequently resealing or reconfiguring the items in the event of process failure is also practical. On the other hand, no volatile organic compounds are released during microwave exposure, ensuring a safer work experience. A case study upon the efficiency of using microwave technology, with a brief cost analysis emphasizing the impact of cost elements, such as energy, processing speed, product quality, equipment cost vs. power, operating cost, and payback period, is presented in [47], concluding that the average repayment period is about four times shorter compared to conventional thermal process.

Ultimately, the final assessment would focus on the recyclability of these hot melts within the framework of the circular economy. As previously stated, the manufacture of MW-activatable adhesives utilized polyethylene matrix from recycled materials, a topic extensively covered in [48,49,50] as regards the sources and specific characteristics of such matrices. In contrast to commonly used, mostly non-recyclable hot-melt adhesives, our suggested composites can be recycled entirely using microwaves to separate the materials and gather them for future use. Accordingly, the recycling procedure includes item sorting and dismantling, hot melt gathering, and grinding. However, it should be noted that recycling hot-melt adhesives does not imply their immediate reuse after the recycling process. The obtained powder is meant to be incorporated into new hot melts in a ratio of up to 25%. The process relies solely on extrusion to create materials like films for new bonding processes. As long as LDPE reprocessing through extrusion is widely known, recycled materials can be reprocessed right away using the same extrusion method, possibly with the addition of small amounts of additives to complete their formula. This innovation entails developing eco-friendly hot-melt adhesive formulations that can be completely recycled and reused for similar bonding requirements. Additionally, using reversible bonding techniques with microwave energy is advantageous, especially in the automotive and construction industries. The bonding process is carried out in industrial settings where the temperature must not surpass 30 °C and the humidity is strictly controlled, where the robotization process efficiently and economically integrates the heating equipment using microwave energy, which offers reduced size, increased dependability, and energy-efficient characteristics. Recycling hot-melt adhesives is crucial in these areas, as European regulations mandate thorough and specific recycling of all related components or materials [1,51].

## 4. Conclusions

This study presents the development of composites using recycled LDPE and 7.5% nanofillers of Al and Fe in two varying particle sizes (800 nm and 50 nm) to be potentially used as hot-melt adhesives for reversible bonding processes with the use of microwave energy. Microwave bonding provides significant advantages over conventional thermal polymer-to-polymer bonding techniques. The heating process begins from the core to the surface of the hot melt and is uniform, not affecting the materials being bonded, including aspects like expansion or internal mechanical stresses. The energy usage is significantly lowered since only the hot melt is heated in an inefficient manner.

As regards the mechanical features of these hot melts, the rise in mechanical resistance is more significant, whereas the elongation is reduced in the Fe-based composites, particularly in those with smaller particles that are distributed more uniformly. Regarding flow resistance, materials with larger particle sizes are preferred, and the metal type has a minimal effect on flow resistance.

Concerning water absorption, it was noted that the swelling degree stayed minimal until 400 h of immersion, after which it rapidly escalated until achieving a saturation point at approximately 600 h. The extent of swelling was higher in the composites containing Al and in those with smaller particles due to the presence of more micro-voids within the materials. Following 400 h of immersion, the composites with smaller particle sizes displayed a particular occurrence where the degree of swelling seemed to be almost the same, irrespective of the type of metallic particle involved. Regarding the extent of swelling with toluene, it was noted that due to toluene’s affinity for the matrix, the values increased, even with briefer immersion durations. The composites containing Al experienced more swelling, as did those with finer particles. In the case of the solvent, saturation occurred earlier, at approximately 450 h, and the extent of swelling was comparable across all the composites, indicating that the absorption of toluene is not significantly influenced by the quantity or dimensions of micro-voids. An interesting finding was noted regarding the final swelling saturation levels in water and toluene, which were almost the same, of approximately 11%, suggesting a complete filling of micro-voids after an immersion time exceeding 500 h.

No matter the metal type, there was a steady rise in dielectric permittivity and dielectric loss factor properties with respect to frequency, starting at 0.8 GHz and reaching a peak approximately at 1.4 GHz for Tan(Delta) in all cases. The size of the particles had little impact, although it seems that compounds with larger particles might exhibit slightly higher parameter values across all the frequency ranges, irrespective of the type of metal. Given that Tan(Delta) plays a crucial role in transforming microwave energy into heat via dielectric loss, the frequency range of 0.9–1.7 GHz is considered optimal for use as the hot melt of these materials. Taking into account all the research on how materials absorb electromagnetic radiation, it is possible to conclude that the best frequency range for maximizing hot melt usage is 0.9–1 GHz. This range is feasible because many industrial/commercial microwave ovens currently operate at 915 MHz, allowing for the development of bonding technologies using these composites as hot melts at the same frequency.

In order to reach the optimal temperature level of 140 °C for bonding using the developed composites, the process requires more than 1.5 min at 570 × 10^3^ W/kg microwave energy and just 0.5 min at 850 × 10^3^ W/kg. As a result, bonding activities require twice as much energy when running at 570 × 10^3^ W/kg, while the efficiency is approximately three times worse compared to operating at 850 × 10^3^ W/kg. For current hot-melt applications, a 30 s exposure is recommended and is comparable to the usual thermal technique, which costs at least ten times more. Finally, it was shown that it is not required to utilize metallic powders smaller than 800 nm or with a restricted dimensional range, or in larger quantities within the composites in this scenario. This offers a substantial cost benefit, since 50 nm sized powders are at least five times more expensive than those sized 800 nm.

The entirety of the presented hot-melt composites can be recycled through microwave technology to separate the elements, enabling them to be collected and reprocessed immediately by a grinding step. The obtained powder is meant to be incorporated into new hot melts in a ratio of up to 25%, to be processed by the same extrusion technique, potentially incorporating minimal additives to enhance their composition for subsequent reuse in similar bonding applications. Furthermore, the utilization of reversible bonding methods with microwave power is beneficial, particularly in the automotive and construction sectors, where the incorporation of heating equipment using microwave energy into the robotization process is efficient and cost-effective, resulting in a smaller size, enhanced reliability, and energy efficiency.

Future research directions will involve experimental data on bonding conditions and bonding resilience under environmental conditions, the recyclability of hot melts, and the reprocessing effect upon the hot melt features.

## Figures and Tables

**Table 1:** Temperature regime for rLDPE (°C).

Heating Zone	5	4	3	2	1
rLDPE—Al	190	186	180	175	170
rLDPE—Fe	195	193	185	180	176

**Table 2:** Recipes’ descriptions (addition in wt%).

Sample Code	Formulation
M1	rLDPE + 7.5% Al/800 nm
M2	rLDPE + 7.5% Al/50 nm
M3	rLDPE + 7.5% Fe/800 nm
M4	rLDPE + 7.5% Fe/50 nm

**Table 3:** Composition of M1.

Formula	Z	Concentration	Most Intense Spectral Line	Statistical Measurement Error	Thickness of the Analyzed Layer
CH_2_	-	92.73%	Organic matter	-	-
Al	13	7.19%	Al KA1-HR-Tr	9.70%	11.8 μm

**Table 4:** Composition of M3.

Formula	Z	Concentration	Most Intense Spectral Line	Statistical Measurement Error	Thickness of the Analyzed Layer
CH_2_	-	92.64%	Organic matter	-	-
Fe	26	7.35%	Fe KA1-HR-Tr	2.06%	0.95 μm

**Table 5:** Results of parameters obtained from microindentation tests.

Sample	H_IT_ (MPa)	HV	E_IT_ (GPa)	S (N/µm)	h_max_ (µm)	W_elastic_ (µJ)	W_plastic_ (µJ)	W_total_ (µJ)	η_IT_ (%)
M1	19.2 ± 0.2	1.8 ± 0.1	0.1 ± 0.01	0.04 ± 0.01	66.9 ± 0.5	16.9 ± 0.1	9.3 ± 0.3	26.1 ± 0.3	65.0 ± 0.6
M2	20.0 ± 1.1	1.9 ± 0.1	0.1 ± 0.02	0.04 ± 0.01	65.4 ± 0.3	16.6 ± 0.3	9.1 ± 0.2	25.7 ± 0.5	64.6 ± 0.2
M3	19.0 ± 0.5	1.8 ± 0.4	0.1 ± 0.02	0.04 ± 0.01	65.3 ± 0.4	15.8 ± 0.2	9.6 ± 0.1	25.4 ± 0.3	62.1 ± 0.2
M4	21.2 ± 1.3	2.0 ± 0.1	0.1 ± 0.01	0.04 ± 0.01	61.0 ± 1.8	14.3 ± 0.4	9.0 ± 0.1	23.3 ± 0.4	61.2 ± 0.5

**Table 6:** Results obtained from density analysis.

Sample	Density [g/cm^3^]
M1	0.933
M2	0.928
M3	0.960
M4	0.937

**Table 7:** Results obtained from shore hardness tests.

Sample	Shore Hardness [MPa]
M1	58
M2	64
M3	59
M4	69

**Table 8:** Experimental data obtained for the mechanical tests.

Sample	Mechanical Resistance [MPa]	Flow Resistance [MPa]	Elongation [%]	Young’s Modulus [GPa]
M1	5.12	0.17	133	0.01
M2	7.42	0.14	100	0.03
M3	6.74	0.18	104	0.01
M4	8.93	0.15	96	0.03

**Table 9:** Degree of swelling in water, at different immersion times [%].

Sample	72 h	168 h	240 h	336 h	408 h	504 h	576 h
M1	4.4147	4.5314	5.2729	5.8699	7.3488	8.7460	8.7460
M2	6.1095	6.8577	7.1886	7.5236	8.3946	10.9999	10.9999
M3	0.6470	2.2700	2.6299	4.8786	6.1141	6.6090	6.6095
M4	1.9535	2.8318	3.8943	6.0172	8.0319	11.0827	11.0844

**Table 10:** Degree of swelling in toluene, at different immersion times [%].

Sample	72 h	168 h	240 h	336 h	408 h	504 h	576 h
M1	7.9868	8.6907	8.8797	9.5472	10.4701	10.7932	10.8056
M2	8.1148	8.8822	9.5250	9.6802	10.5641	10.9785	10.9908
M3	8.2854	9.3585	9.5383	10.2608	10.9119	11.0490	11.0613
M4	9.1654	9.6462	9.9119	10.7267	11.0407	11.1217	11.1341

**Table 11:** Determined DSC parameters.

Sample	T_t_ (°C)	ΔH_t_ (J/g)	χ_cr_ (%)	OOT_1_ (°C)	OOT_2_ (°C)
M1	103.7	40.1	14.9	212	315
M2	104.5	36.4	13.5	209	307
M3	102.9	46.3	17.2	204	251
M4	104.5	40.1	14.3	188	239

**Table 12:** Experimental data obtained for the bonding process.

Bonded Items	Hot Melt Sample	Mechanical Resistance [MPa]	Elongation [%]
LDPE + LDPE	M1	11.38	33
LDPE + HDPE	M1	10.84	28
LDPE + PP	M1	11.11	31
HDPE + PP	M1	10.75	25
PP + PP	M1	10.31	21
LDPE + LDPE	M3	11.12	32
LDPE + HDPE	M3	10.78	27
LDPE + PP	M3	10.93	28
HDPE + PP	M3	10.83	24
PP + PP	M3	10.68	21

## Data Availability

The data are presented in this study.
